# Polarization-probe polarization-imaging system in near-infrared regime using a polarization grating

**DOI:** 10.1038/s41598-022-19536-3

**Published:** 2022-09-10

**Authors:** Moritsugu Sakamoto, Huynh Thanh Nhan, Kohei Noda, Tomoyuki Sasaki, Masayuki Tanaka, Nobuhiro Kawatsuki, Hiroshi Ono

**Affiliations:** 1 Department of Electrical, Electronics, and Information Engineering, Nagaoka University of Technology, 1603-1 Kamitomioka, Nagaoka, Niigata 940-2188 Japan; 2 OPT Gate Co., Ltd, 1-16-12, Shibamata, Katsushika-ku, Tokyo, 125-0052 Japan; 3 Department of Applied Chemistry, University of Hyogo, 2167 Shosha, Himeji, Hyogo 671-2280 Japan; 4 CREST, Japan Science and Technology Agency, Chiyoda-ku, Tokyo 102-0076 Japan

**Keywords:** Imaging and sensing, Electrical and electronic engineering

## Abstract

A polarization-probe polarization-imaging (PPPI) system was developed for the near-infrared (NIR) regime. This system comprises two components operating as a polarization generator and a polarization analyzer to enable polarization image capture under polarized light illumination. The captured polarization images contain considerable object information because the illuminating polarized light beams are affected by many of the Mueller matrix elements. By assembling the polarization camera using two liquid crystal retarders and a polarization grating, the PPPI system offers the potential to measure the Stokes parameters fully with a high extinction ratio, even in the NIR region. The PPPI system’s feasibility was demonstrated experimentally. Its dependence on the state of polarization (SoP) of the illuminating polarized light was discussed. The polarization image acquired by the PPPI system is strongly dependent on the illuminating light’s SoP, so the appropriate SoP must be selected for each object to enhance the polarization image contrast. This PPPI system should expand the range of polarization imaging applications, including LiDAR, product inspection, and bio-imaging.

## Introduction

Polarization imaging is currently an important topic in the optical sensing field because the state of polarization (SoP) of a light beam that is scattered by an object contains a variety of information about the object’s surface structure and anisotropy. Therefore, many potential applications of polarization imaging have been proposed, including biomedical sensing and remote sensing^1–5^. To measure these polarization images, various approaches have been proposed to date, including methods based on a rotating polarizer/compensator polarimeter, a micro-polarizer/retarder array camera^6–11^, a channeled imaging polarimeter^12–16^, and an amplitude division camera^17–21^. Some polarization imaging methods are already being incorporated into commercial products.

To enable advancement of the modern trend for automatic control of hardware, polarization imaging in both the visible regime and the near-infrared (NIR) regime should provide a powerful visual sensing tool because NIR light has numerous merits from an optical sensing viewpoint, including eye safety, its ability to pass through opaque materials, including its ability to penetrate human tissue, and independence from the color distribution of objects of interest. Therefore, in recent years, polarization imaging in the NIR region has become a research hotspot^22–26^. To perform polarization imaging in the NIR regime, a rotating polarizer^26^ and a micro-polarizer array camera^23,25^ have been used. However, the method based on the rotating polarizer requires use of a mechanical element for the rotation, and considerable care must therefore be taken to ensure stable operation. In addition, the micro-polarizer array camera shows a relatively low extinction ratio (ER) for NIR light because of the influence of diffraction from the array boundary, which causes crosstalk among neighboring pixels. A high ER is an important factor in polarization imaging because it is related to the measurement accuracy. To address this requirement, Maruyama et al. reported a four-directional on-chip polarization-type complementary metal-oxide-semiconductor (CMOS) image sensor that can improve the ER by closing the polarizers to the photodetectors in the visible regime^11^, although there is no report that this device was adapted for use in the NIR regime. Additionally, the micro-polarizer array camera cannot measure the $S_3$ parameter corresponding to the intensity of the circular polarization components. In our previous work, we also proposed a polarization imaging method that used a liquid crystal polarization grating (LCPG)^19^ as an amplitude division camera. This method can obtain a full Stokes parameter image of an object of interest without any requirement for moving mechanical parts, in contrast to methods that use a rotating polarizer/compensator. In addition, unlike the method based on the micro-polarizer array, which is a pixel division-type approach, no crosstalk occurs between the pixels because of diffraction. Therefore, even if our method is used with a light source in the NIR regime, it offers the advantage of providing a superior ER. Additionally, this method can measure all the Stokes parameters, including $S_3$.

**Figure 1:**
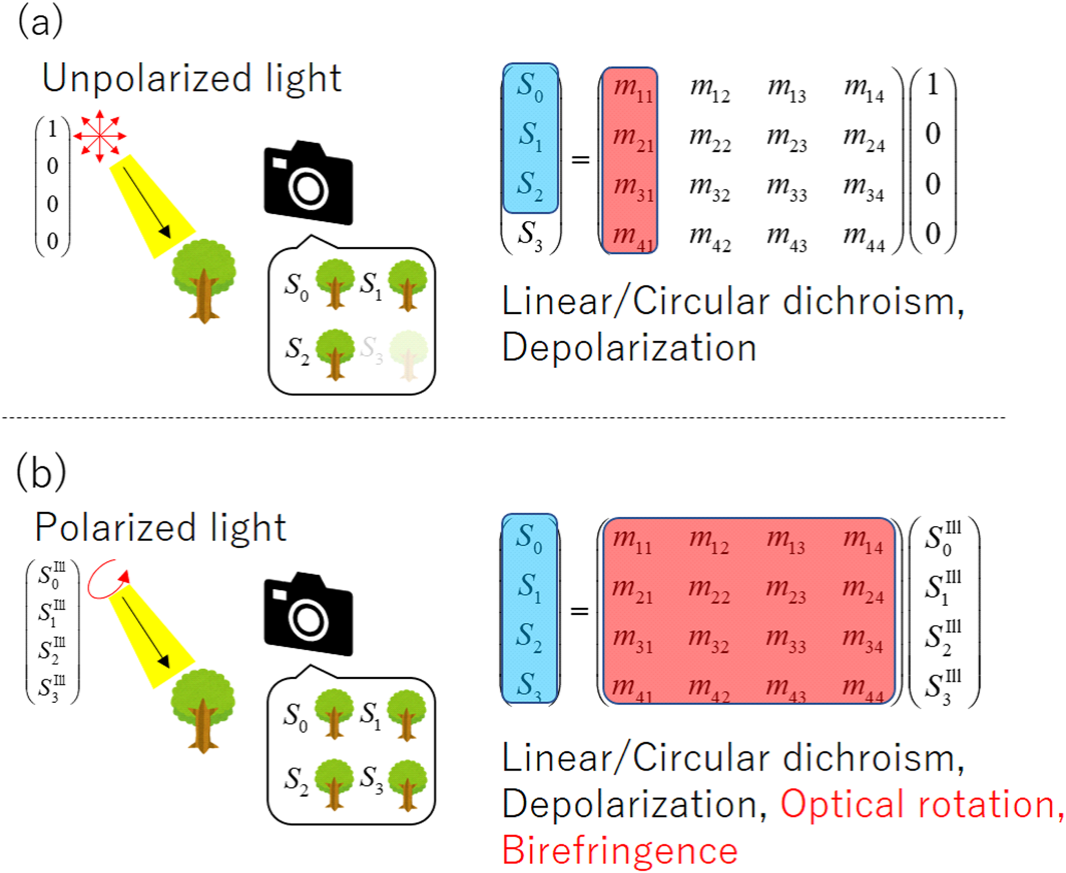
Description of polarization conversion based on the Muller matrix formula. (**a**) Conventional approach for measurement of the Stokes parameters $S_0$, $S_1$, and $S_2$ under unpolarized light illumination whose Stokes vector is $\left( 1, 0, 0, 0\right)$. (**b**) PPPI approach for measurement of the Stokes parameters $S_0$, $S_1$, $S_2$, and $S_3$ under polarized light illumination whose Stokes vector is $\left( S_0^{\text{Ill}}, S_1^{\text{Ill}}, S_2^{\text{Ill}}, S_3^{\text{Ill}}\right)$.

**Figure 2:**
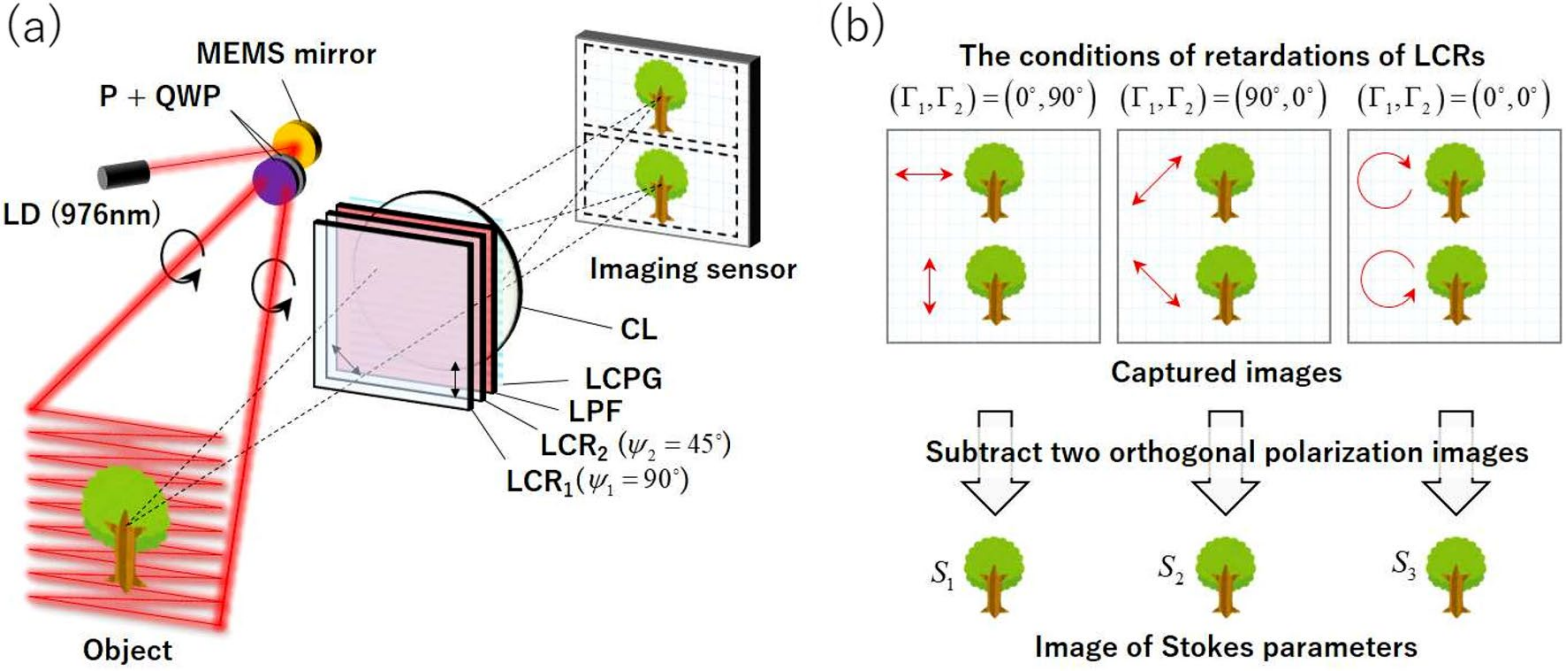
(**a**) Schematic of the PPPI system using the liquid crystal polarization grating (LCPG). The PPPI system consists of a polarization generator and a polarization analyzer. The polarization projector is assembled from the near-infrared-laser diode (NIR-LD), the micro-electro-mechanical systems (MEMS) mirror, the polarizer (P), and the quarter-wave plate (QWP). The polarization camera is assembled from the liquid crystal retarders (LCR$_1$ and LCR$_2$), the long-pass filter (LPF), the LCPG, the camera lens (CL), and the imaging sensor. The $\psi _1$ and $\psi _2$ are the orientation angles of fast axis of LCRs. (**b**) Principle of PI using the LCPG and two LCRs.

**Figure 3:**
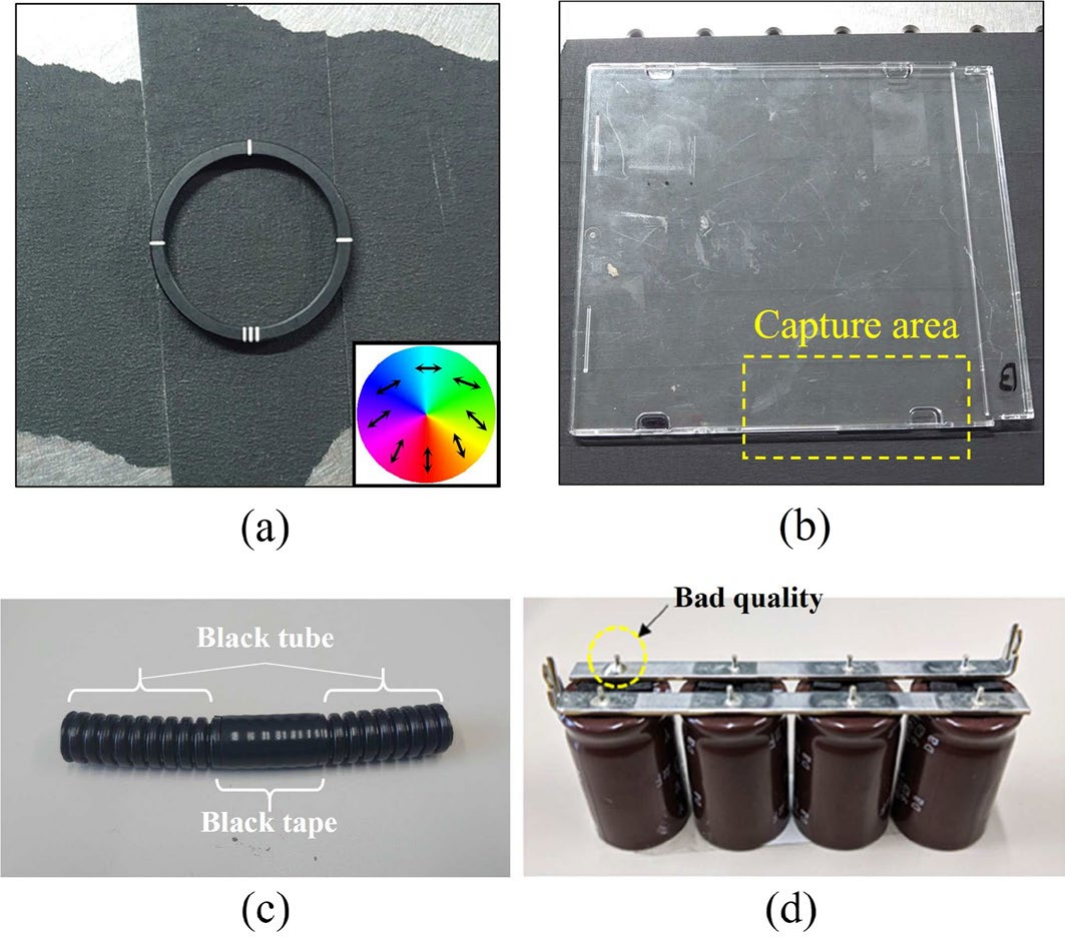
(**a**–**d**) the prepared samples. (**a**) Patterned retarder with a fast axis distribution that is illustrated on the lower right of the photograph. (**b**) CD case. (**c**) Black tube wrapped in black tape. (**d**) Soldered condenser.

**Figure 4:**
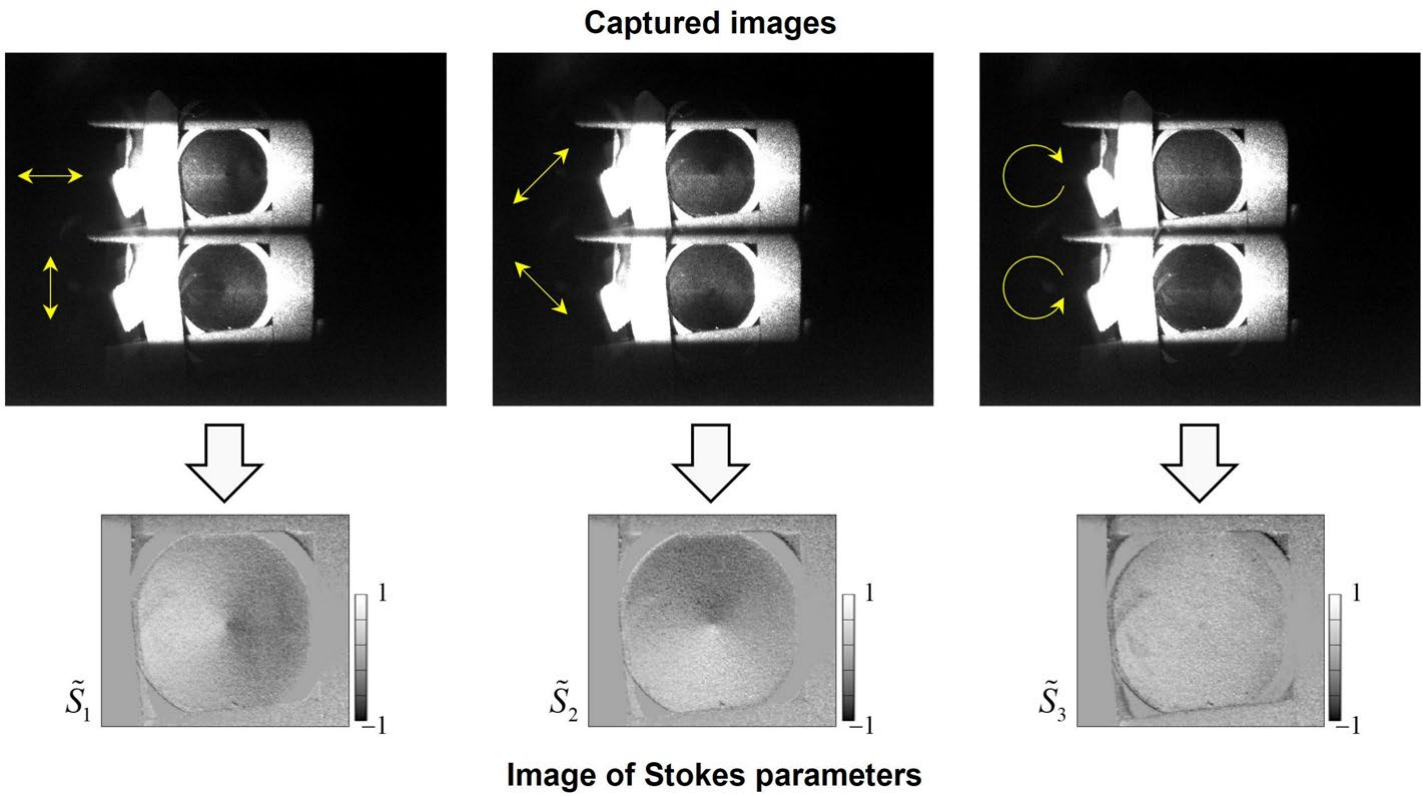
Captured images and reconstructed Stokes parameters of the patterned retarder where the sample was illuminated with RCP light.

A change in the Stokes vector can be written using the Mueller matrix formula. Figure 1 shows a schematic of the polarization imaging method and its relationship with the Mueller matrix. The 16 elements of the Mueller matrix contain many different types of object information, including linear/circular dichroism, birefringence, optical activity, and depolarization data^27^. Therefore, to reveal the object information in detail, it is desirable to measure the Stokes vector when affected by all these elements. However, in many existing polarization imaging methods, the object is illuminated with unpolarized light (UPL), particularly in outdoor demonstrations. Also, measuring Stokes parameters are limitted to $S_0$, $S_1$, and $S_2$. In this case, the sensitivities to both the birefringence and the optical activity are lost, thus making it difficult to obtain the polarization characteristics of the object accurately from the captured polarization image. Therefore, to enhance the effects of polarization imaging further, it is desirable to be able to measure the Stokes parameters in full under polarized light illumination conditions. Under these circumstances, we have developed a polarization-probe polarization-imaging (PPPI) system that offers both polarized light illumination and full Stokes parameter imaging functions. This system makes it possible to acquire a variety of information remotely, including the internal optical anisotropy and surface fine structure properties of objects. Using the advantages of the polarization imaging method based on use of the LCPG, we demonstrate PPPI in the NIR regime for several object types.

## Results

### Schematic of a PPPI system using LCPG

Figure 2a shows a schematic of the PPPI system using the LCPG. This system consists of an NIR laser diode (NIR-LD), a microelectromechanical system (MEMS) mirror, a polarizer (P), a quarter-wave plate (QWP), two liquid crystal retarders (LCR$_1$ and LCR$_2$), a long-pass filter (LPF), the LCPG, a camera lens (CL), and an imaging sensor. In this system, a laser beam that is output from the NIR laser is first reflected by the MEMS mirror and then scanned onto the object plane along with a raster orbit. The SoP of the scanning laser beam is controlled using a set composed of the P and the QWP. The scattered and reflected light from the object is then passed successively through the LCRs, the LPF, the LCPG, and the CL. The LCPG has a function that allows it to diffract the right- and left-handed circular polarizations (RCP and LCP) selectively to the +1st and -1st order directions because of the effect of the Pancharatnum-Berry phase^28–32^. The set composed of LCR$_1$ and LCR$_2$ acts as a polarization converter, converting 0 deg and 90 deg (45 deg and -45 deg) linear polarization (LP) components into RCP and LCP components when the retardations are set at $\Gamma _1$ = 90 deg and $\Gamma _2$ = 0 deg ($\Gamma _1$ = 0 deg and $\Gamma _2$ = 90 deg), respectively. The $\psi _1=90$ deg and $\psi _2=45$ deg are the orientation angles of fast axis of LCRs. An object is imaged on the imaging sensor using a CL inserted between the LCPG and the imaging sensor. By appropriate selection of the retardations of the LCRs, images of six polarization states were obtained from the three captured images, as illustrated in Fig. 2b. As a result, the images of the Stokes parameters of the object can be obtained via subtraction of two orthogonal polarization images. This principle was described in detail in our previous work^19^. Note here that the laser scan pattern width in the split direction should be limited to less than half of the field of view in the split direction of the imaging system to avoid any overlap between the two orthogonal polarization images that were split using the LCPG. Experimentally assembled PPPI system is described in "Methods" section.

### Prepared samples for the demonstration of PPPI

To demonstrate the performance of the PPPI system, we prepared a patterned retarder made from polymerized liquid crystal (WPV10L-405; Thorlabs Inc.), a compact disc (CD) case, a black tube wrapped with black tape, and a soldered condenser as sample objects. Photographs of these samples are shown in Fig. 3a–d, respectively. The prepared patterned retarder has a space-variant fast axis distribution on which the retardation has a uniform value. The prepared CD case contains distortion such that it also shows anisotropy in the same manner as the patterned retarder. We expected the PPPI system to visualize the anisotropy patterns of these samples. The prepared tube sample contained two different components with the same black color. We expected these two parts to be identified by the PPPI system. The prepared soldered condenser has eight soldered parts, including one bad quality part [see the dashed yellow circle in Fig. 3d] and seven good quality parts. We also expected to be able to detect the bad quality soldered part from the PI.

### Spatially divided images of orthogonal polarization components obtained by the PPPI system

**Figure 5:**
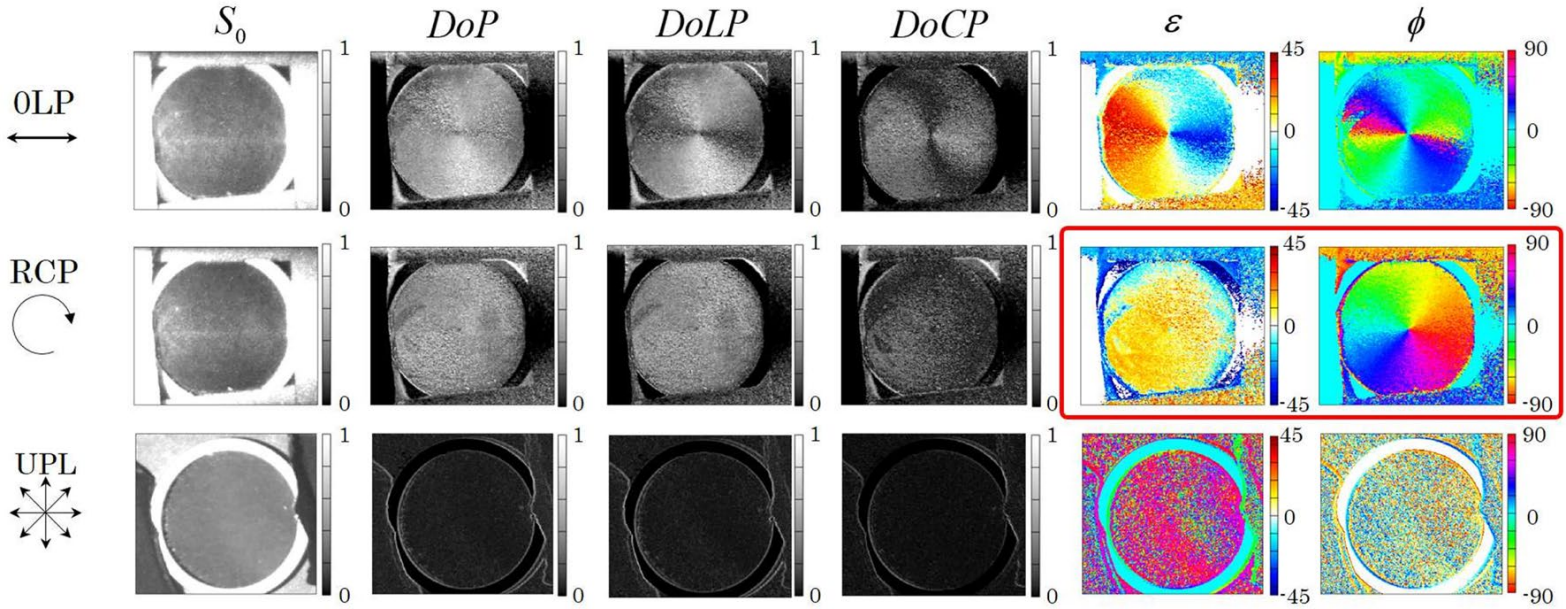
Intensity, degree of polarization, degree of linear polarization, degree of circular polarization, ellipticity angle, and azimuth angle properties calculated from the Stokes parameters of the patterned retarder obtained using the PPPI system.

**Figure 6:**
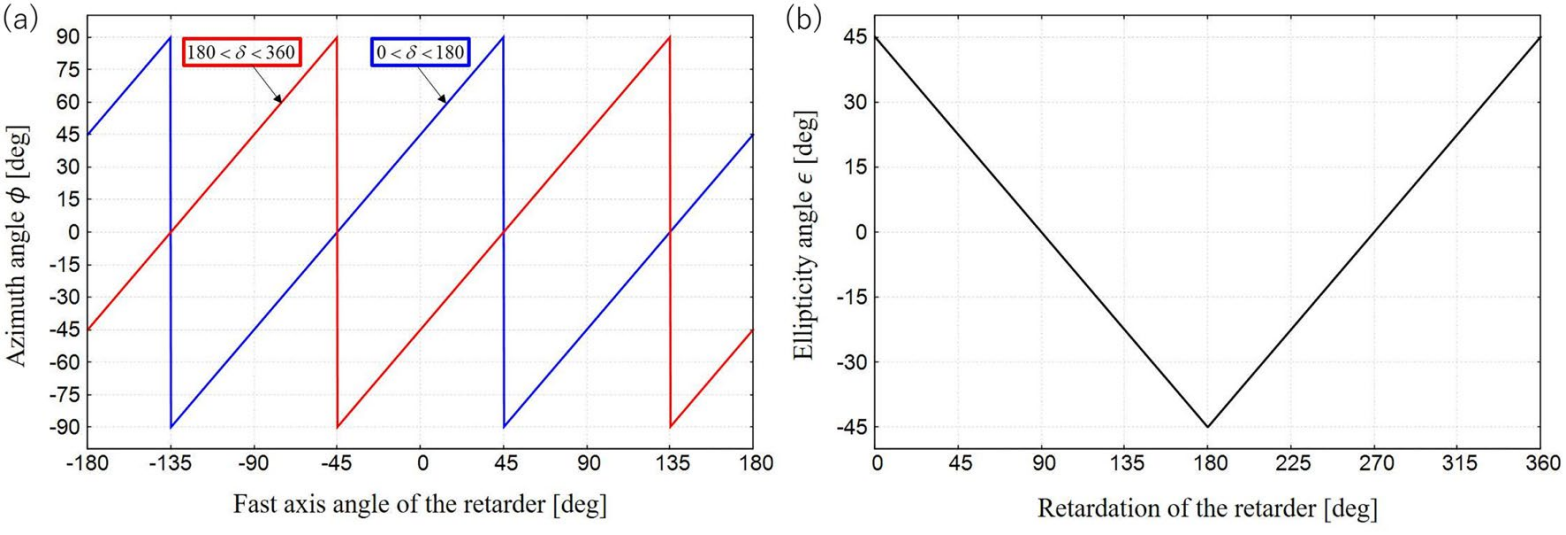
Numerically calculated (**a**) azimuth and (**b**) ellipticity angle plotted as functions of fast axis and retardation of the retarder.

**Figure 7:**
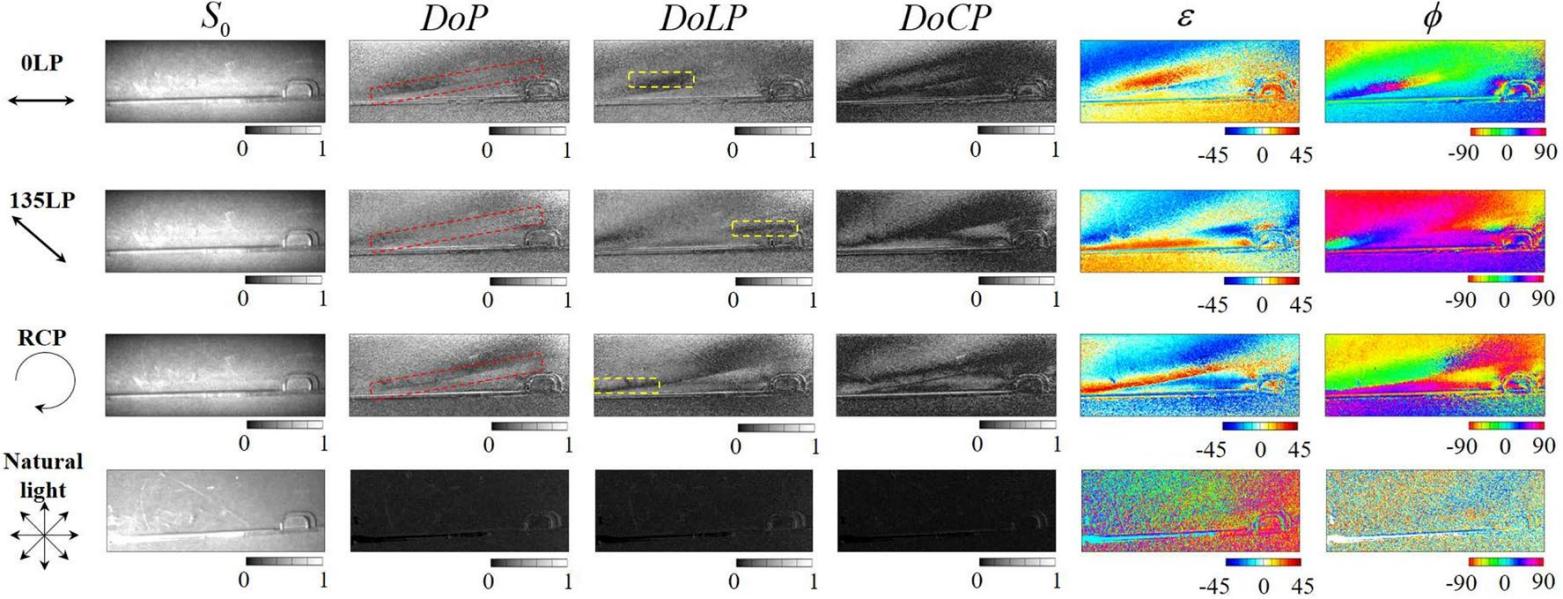
Intensity, degree of polarization, degree of linear polarization, degree of circular polarization, ellipticity angle, and azimuth angle calculated from the Stokes parameters of the CD case obtained using the PPPI system.

**Figure 8:**
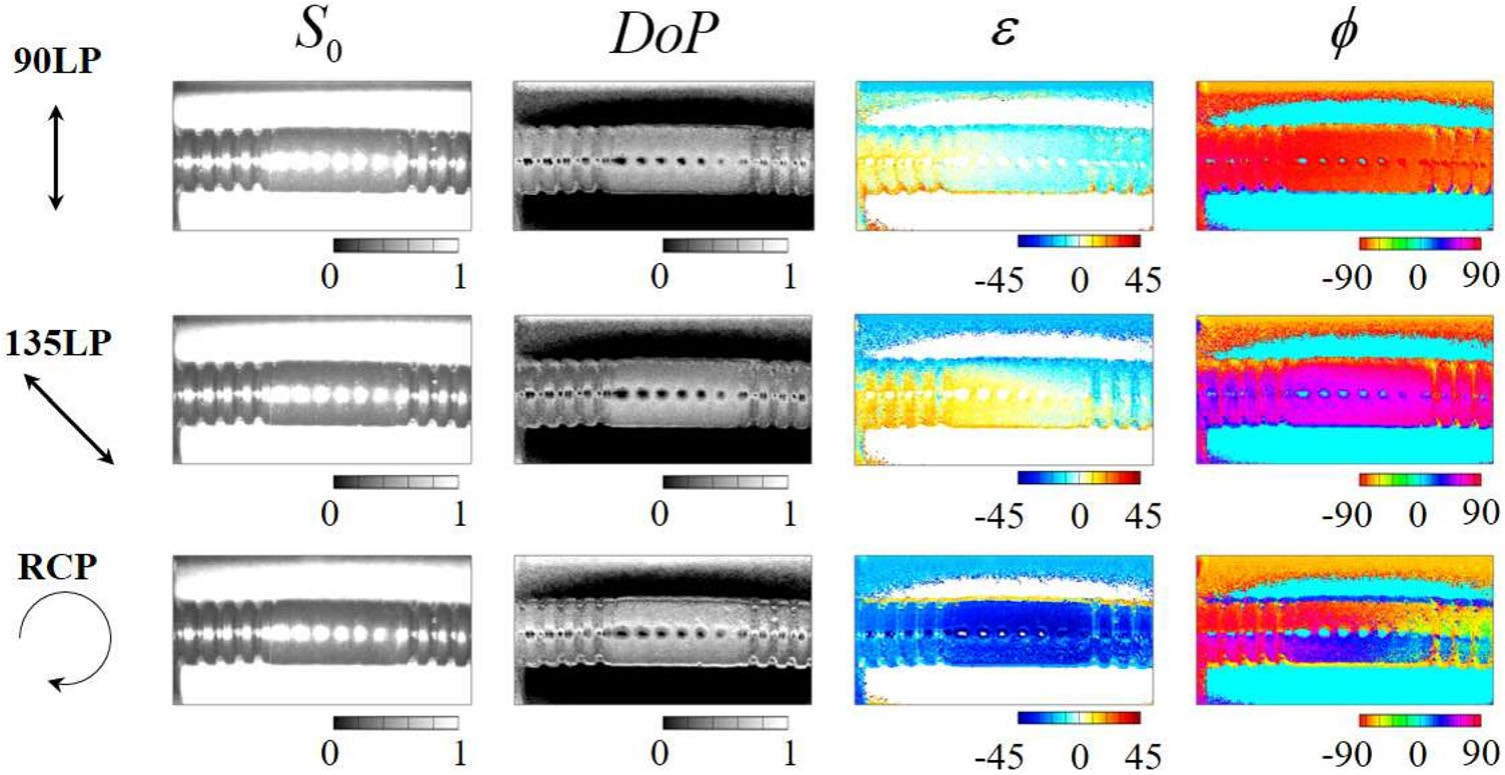
Intensity, degree of polarization, ellipticity angle, and azimuth angle calculated from the Stokes parameters of a black tube wrapped in black tape that were acquired using the PPPI system.

The upper row of photographs in Fig. 4 shows the three captured images of the patterned retarder that were acquired by changing the retardations of the LCRs. The SoP of the illumination light was set to be RCP. The images of the 0, 90, 45, and -45 deg LP, RCP, and LCP components were obtained in three captured images. From each captured image, the images of the Stokes parameters were analyzed by subtracting the orthogonal SoP image pixel by pixel. These parameters were normalized using an $S_0$ image that was reconstructed by summing of orthogonal SoP images. The reconstructed normalized Stokes parameters ${\tilde{S}}_1=S_1/S_0$, ${\tilde{S}}_2=S_2/S_0$, and ${\tilde{S}}_3=S_3/S_0$ are shown in the lower row of images in Fig. 4. Using ${\tilde{S}}_1$ and ${\tilde{S}}_2$, the space-variant SoP pattern was captured by the PPPI system. To allow the captured SoP pattern to be analyzed in detail, we then calculated the degree of polarization (DoP), the degree of linear polarization (DoLP), the degree of circular polarization (DoCP), the ellipticity angle, and the azimuth angle using the reconstructed normalized Stokes parameters. These parameters are defined as:

1 $$\begin{aligned} P= {} \sqrt{{\tilde{S}}^2_1+{\tilde{S}}^2_2+{\tilde{S}}^2_3}, \end{aligned}$$

2 $$\begin{aligned} P_{\text{L}}= {} \sqrt{{\tilde{S}}^2_1+{\tilde{S}}^2_2}, \end{aligned}$$

3 $$\begin{aligned} P_{\text{C}}= {} \sqrt{{\tilde{S}}^2_3}, \end{aligned}$$

4 $$\begin{aligned} \varepsilon= {} \frac{1}{2}\tan ^{-1}\left( \frac{{\tilde{S}}_3}{\sqrt{{\tilde{S}}^2_1+{\tilde{S}}^2_2}}\right) , \end{aligned}$$

5 $$\begin{aligned} \phi= {} \frac{1}{2}\tan ^{-1}\left( \frac{{\tilde{S}}_2}{{\tilde{S}}_1}\right) , \end{aligned}$$

where *P*, $P_{\text{L}}$, $P_{\text{C}}$, $\varepsilon$, and $\phi$ are the DoP, DoLP, DoCP, ellipticity angle, and azimuth angle, respectively. The results are shown in Fig. 5. For the polarized light illumination (0LP and RCP) case, both $\varepsilon$ and $\phi$ showed space-variant values that corresponded to the retardation and the fast axis of the patterned retarder. In contrast, no pattern was obtained in the unpolarized light illumination case. The results of polarization analysis for the samples of Fig. 3b–c are also shown in Figs. 6, 7 and 8, respectively.

## Discussion

We focus on Fig. 5 to enable discussion of the effectiveness of the PPPI system. The prepared patterned retarder was a wave plate in which the optical axis was rotated continuously around the center of the element. From the results shown in Fig. 5, the three types of polarization illumination (0LP, RCP, and unpolarized light) have the following characteristics. First, in the case of 0LP illumination, because the ellipticity and the azimuth angle change according to the object’s anisotropy, it is possible to visualize information about the spatial distribution of this anisotropy. However, it is not possible to distinguish between the fast and slow axes of the anisotropy, and the polarization change sensitivity is lost when the polarization azimuth of the illuminated LP component is oriented either parallel or orthogonal to the optical axis. Then, in the RCP illumination case, the direction and the magnitude of the anisotropy of the object can be visualized from $\phi$ and $\varepsilon$, respectively. The polarization azimuth is proportional to the asymmetric optical axis azimuth, and the all-optical axis azimuths are sensitive, which means that the azimuth of the anisotropic spatial distribution can be restored accurately. Specifically, the azimuth angle obtained is equal to a pattern that is rotated with respect to the original fast axis distribution by 45 deg. In contrast, in the unpolarized light illumination case, the SoP of the light is insensitive to the anisotropy. Here, we quantitatively discuss the change of $\phi$ and $\varepsilon$ from patterned retarder. In the case of our experiment, the illuminating RCP first passes through the patterned retarder. The output light then again passed through the patterned retarder due to the back scattering at the black paper. As a result, the illuminating light suffers double the value of retardation of patterned retarder. When an RCP passes through the retarder whose fast axis are aligned at angle of $\psi$, a Stokes vector of output beam can be written as

6 $$\begin{aligned} \left[ \begin{array}{c} S_0 \\ S_1 \\ S_2 \\ S_3 \\ \end{array} \right] = \left[ \begin{array}{c} 1 \\ \sin 2\psi \sin \delta \\ -\cos 2\psi \sin \delta \\ \cos \delta \\ \end{array} \right] , \end{aligned}$$

where $\delta$ is the retardation of the retarder. From these parameters, we can numerically calculate the azimuth and ellipticity angles using eqs. (4) and (5). Figure 6a shows azimuth angle plotted as functions of the fast axis of the retarder. From this graph, azimuth angle can be written as

7 $$\begin{aligned} \phi = {\left\{ \begin{array}{ll} \psi -45 &{} \text{if}\, 0< \delta< 180, \\ \psi +45 &{} \text{if} \,180< \delta < 360. \end{array}\right. } \end{aligned}$$

From eq. (7), we found that the azimuth angle corresponds to the fast axis angle of retarder. A bias from the fast axis is ±45 deg, whose sign of bias depends on the magnitude of retardation. On the other hand, Fig. 6b shows ellipticity angle plotted as functions of retardation of the retarder. From this graph, ellipticity angle can be written as

8 $$\begin{aligned} \varepsilon = {\left\{ \begin{array}{ll} 45-\frac{\delta }{2} &{} \text{if} \,0 \le< \delta< 180,\\ \frac{\delta }{2}-135 &{} \text{if} \,180 \le \delta < 360. \end{array}\right. } \end{aligned}$$

From eq. (8), we found that the ellipticity angle corresponds to the retardation of retarder. The ellipticity angle is independent of the fast axis of retarder. Also, equations should be selected depending on the magnitude of retardation. Therefore, a pair of $\phi$ and $\varepsilon$ obtained by circularly polarized light illumination is the appropriate choice to enable visualization of the principal axis and the birefringence pattern of anisotropic objects as indicated by red flame in Fig. 5.

**Figure 9:**
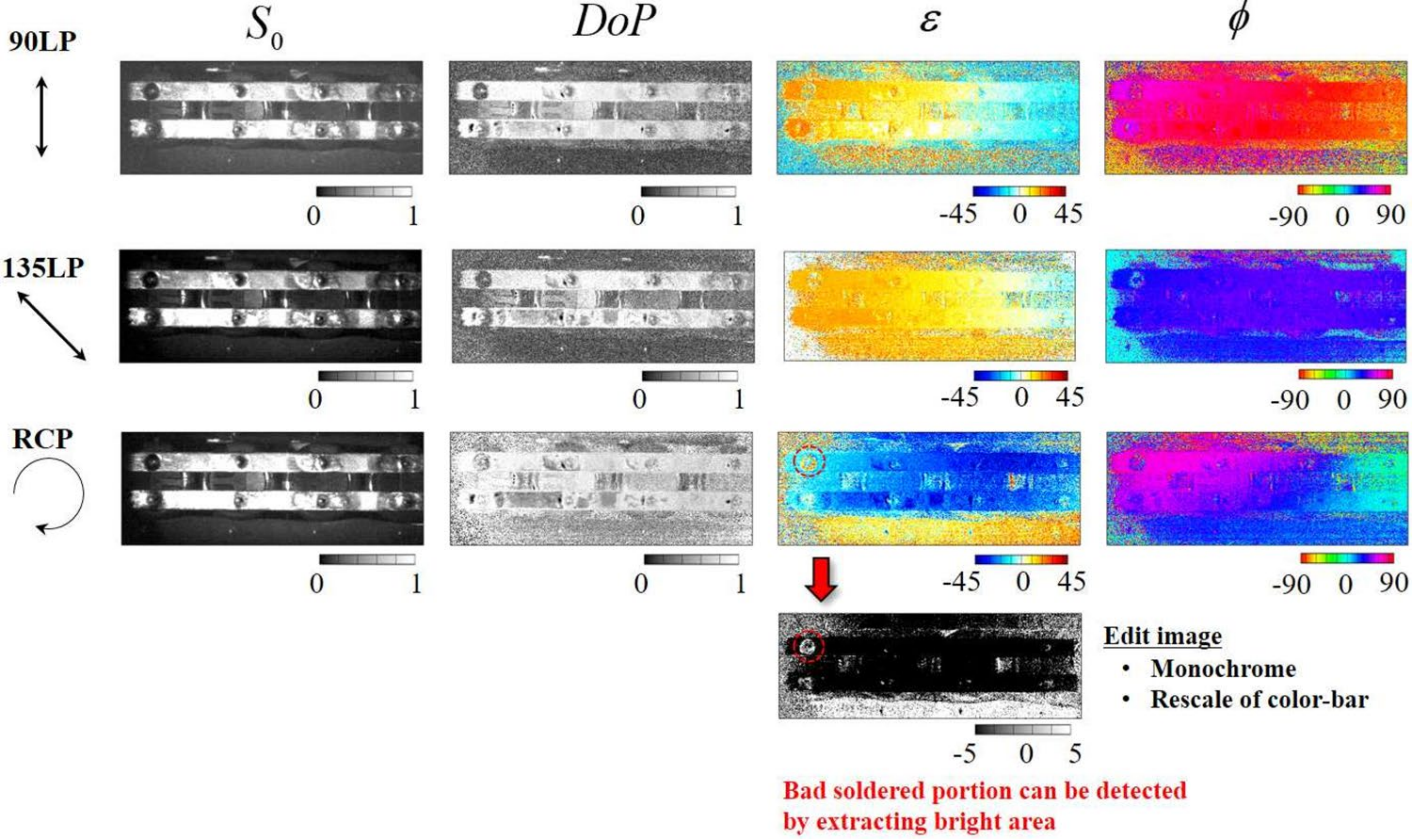
Intensity, degree of polarization, ellipticity angle, and azimuth angle calculated from the Stokes parameters of the soldered condenser that were acquired using the PPPI system.

With regard to Fig. 5, we also consider the importance of measurement of all the Stokes parameters here, including $S_3$. In case where only $S_0$, $S_1$, and $S_2$ are measured, we can then only reconstruct $S_0$, $P_{\text{L}}$, and $\phi$. Because the DoLP is missing the $S_3$ information, the $P_{\text{L}}$ is then reduced when a circularly polarized light component is included within the captured image. This means that the presence of anisotropy causes deterioration of the DoLP. As a result, it is impossible to distinguish between the optical anisotropy and pure depolarization caused by scattering. In addition, the magnitude of the anisotropy cannot be visualized in this case because $\varepsilon$ cannot be reconstructed. In contrast, the DoP is immune to ovalization of the SoP, which means that it can visualize the pure depolarization obtained correctly. Therefore, measurement of the full Stokes parameters is essential to enable precise visualization of the object information.

Figure 7 shows polarization images of a CD case acquired using the PPPI system. As described above, $\varepsilon$ and $\phi$ represent the magnitude and direction of the anisotropy and indicate that the prepared CD case has spatially distributed anisotropy caused by internal distortion. The value of the $P_{\text{L}}$ decreases in the area containing the circularly polarized light components, which is indicated by the yellow frame. In contrast, we can find a line shape region in which the *P* has a low value, as indicated by the red frames. Because this region shows differences in deterioration between the *DoP* and the $P_{\text{L}}$, this low *P* line cannot be explained by the images containing the circularly polarized light component. The reason for this deterioration can be considered to be due to the scattered structure and the steep space-variant polarization distribution. When the SoP is changed spatially and steeply, with a modulation period that is equal to or smaller than the single pixel size of the imaging sensor, the single pixel receives several SoP components simultaneously. This causes depolarization within the single pixel because the depolarization corresponds to the temporal and spatial uniformity of the SoP. Because the scattering structure cannot be determined from the $S_0$ image, we consider this deterioration to be caused by a steep polarization change within the single pixel. Additionally, we also consider the dependence on the SoP of the illuminating light. In the case of illumination with 135 deg linear polarization (135LP), the line-shaped deterioration of the DoP is not observed within the red framed region, whereas depolarizations are observed in the 0LP and RCP illumination cases. The principal axis of anisotropy caused by distortion can be considered to be parallel or perpendicular to the 135 deg direction, which means that the depolarization due to the steep spatial polarization change does not occur in the 135LP illumination case. As described in the results for the patterned retarder, because the sensitivity to polarization change is lost when the polarization azimuth of the illuminated LP component lies parallel or orthogonal to the optical axis, use of circularly polarized light as the illumination light for the PPPI system is preferred to enable visualization of the inner distortion of an object from the images of *P*, $\varepsilon$, and $\phi$, for which the principal axis pattern is unknown.

Additionally, we present the results of PPPI of industrial products (including a black tube wrapped in black tape [Fig. 3c] and a soldered condenser [Fig. 3d]) in Figs. 8 and 9, respectively. From the image in Fig. 3c, it is not easy to identify the differences between the tube and the wrapped tape from the normal visible photograph because these parts have the same shade of black. In contrast, from Fig. 8, when the RCP illumination is incident on the sample, we found that the polarization images allowed us to distinguish between the black tube portion and wrapped tape portion of the same color from the image of the DoP and ellipticity angle. The black tube part shows a periodic uneven shape, whereas the black tape part shows a smooth shape; we therefore consider that a difference in the polarization conversion that is dependent on scattering and Fresnel reflection appears in this case. Then, we focused on the results for the soldered condenser. From Fig. 3d, it is not easy to detect the bad quality soldered area. In contrast, from the $\varepsilon$ image in Fig. 9 [see the red circle position], we also found that the bad quality soldered area could be detected because $\varepsilon$ shows opposite signs for the bad and good quality areas. To enhance the contrast of image, we replot the $\varepsilon$ image as monochrome map with rescaling color-bar. We found that bad soldered portion can be detected by extracting bright area from the $\varepsilon$ image. This means that rescaling of polarization image can extract characteristic point in the captured image. These results indicate that the PPPI system has the potential to be applied to product inspection. In a similar manner to the case of the black tube sample, we consider that this difference in the ellipticity angle is caused by Fresnel reflection, because the bad and good quality soldered areas have different surface shapes to each other. Since surface shape correspond to the spatial incident angle distribution, the SoP of reflected light is spatially distributed due to the difference of Fresnel reflection at each point. In addition, the sign of ellipticity angle is flipped around Brewster’s angle. Based on these properties, Tsuru et al. reported that circularly polarized light illumination can enable derivation of the azimuth and slope angles of the facet of an object via determination of the parameters of the reflected polarization ellipse^33^. However, at this time, we cannot theoretically prove that this consideration is correct, because we do not have an environment for numerical simulation of PPPI. We prepared a computing environment to simulate polarization imaging for various objects under polarized light illumination based on the finite-difference time-domain method, and hence we will report quantitative analysis for PPPI including present samples in future work.

These positive results were obtained in the RCP illumination case, indicating that circularly polarized light illumination should be a powerful tool for use with the PPPI system. In fact, polarized beams have numerous merits for object illumination from an optical sensing viewpoint. For example, circularly polarized light can maintain its degree of polarization longer through larger numbers of scattering events than linearly polarized light in forward-scattering environments^34,35^. Based on this feature, researchers have demonstrated that circularly polarized light illumination and detection under scattering conditions can produce images with higher contrast for objects that maintain their incident degree of polarization^36–38^. Additionally, Tsuru reported that circularly polarized light illumination can enable derivation of the azimuth and slope angles of the facet of an object via determination of the parameters of the reflected polarization ellipse^33^. Besides, Nishizawa et al. reported spatial discrimination of cancer using circular polarized light scattered by biological tissues^39,40^. From these perspectives, circularly polarized beam projection will be useful in polarization imaging. Investigation of PPPI of objects under scattering event conditions will be reported in future work. Also, in view of application, it is important to investigate the difference in PPPI within the range from visible to near-infrared. The wavelength dependency of PPPI for various objects would be reported in future.

## Conclusion

In this study, we have developed a PPPI system for use in the NIR regime. The PPPI system consists of a polarization projector and a polarization camera. The polarization projector component can illuminate an object with rectangular-shaped polarized NIR light. Because the projection pattern is formed by temporal scanning of a laser beam using a MEMS mirror, the captured images are immune to speckle noise. The polarization camera was assembled using two LCRs and a LCPG. The LCPG was made from polymerized liquid crystal and a photocrosslinkable polymer liquid crystal with retardation that was approximately optimized at 976 nm. The feasibility and practicability of the developed PPPI system were demonstrated experimentally for cases including a patterned birefringent plate, a CD case, and industrial products. In the case of the patterned birefringent plate, we confirmed that the direction of the optical axis and its retardation were both visualized using the PPPI system. In the case where the CD case had internal stress, we found that its internal stress pattern could be visualized by the PPPI system. In the cases of both the birefringent plate and the CD case, the use of circularly polarized light for the polarization projection provided the most effective SoP to visualize the anisotropy information of the objects of interest. Additionally, the PPPI system can identify different material portions of an object, even if they are of the same color. Furthermore, the PPPI system showed that a bad quality soldered area could be detected from a captured polarization image. These results indicate that the PPPI system has the potential to be applied to product inspection. Therefore, the PPPI system developed in this work should expand the range of potential applications of polarization imaging to areas including LiDAR, product inspection, and bio-imaging.

## Methods

### Experimentally assembled PPPI system

**Figure 10:**
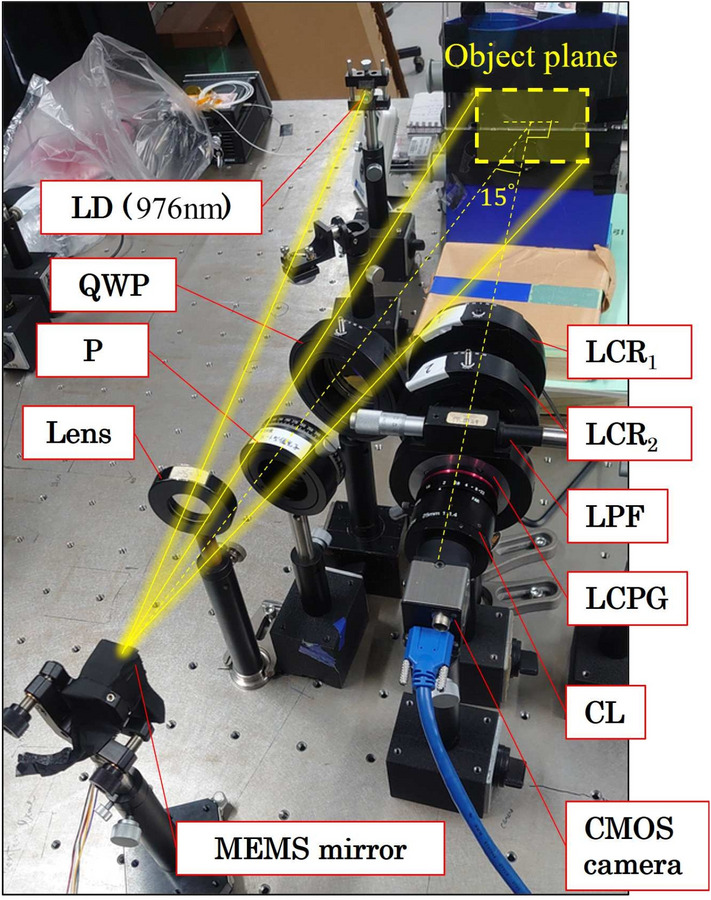
Photographs of the experimentally assembled PPPI system.

A photograph of the experimentally assembled PPPI system is shown in Fig. 10. As the NIR-LD, we used a fiber-Bragg-grating-stabilized LD operating at 976 nm (BL976-SAG300; Thorlabs Inc.). As the image sensor and CL, we used a CMOS camera (DCC3240M; Thorlabs Inc.) and a shortwave infrared (SWIR) fixed focus lens (HS2514V-SW; Myutron Inc.). To eliminate the visible light waves, we used a long-pass filter with a cutoff wavelength of 900 nm (FELH0900; Thorlabs Inc.). As the LCRs, we used two liquid crystal variable retarders with an aperture radius of 20 mm (LCC1223-C; Thorlabs Inc.). We used an LCPG that was fabricated using a photocrosslinkable polymer liquid crystal film^41^ and a polymerized liquid crystal (LC242; BASF Inc.). The retardation of the LCPG was optimized at approximately 976 nm. The measured diffraction efficiency and diffraction angle of the prepared LCPG were 99 % and 2.06 deg, respectively. In view of the use of the circular polarization beam splitter, the ER of the LCPG was defined as $P_{{+1}}=I^{\text{RCP}}_{{+1}}/I^{\text{LCP}}_{{+1}}$ and $P_{{-1}}=I^{\text{LCP}}_{{-1}}/I^{\text{RCP}}_{{-1}}$, where the superscript and the subscript indicate the SoP of light incident on the LCPG and its diffraction order, respectively. According to this definition, we measured the ER of the prepared LCPG to be $P_{{+1}}=294$ and $P_{{-1}}=386$, where the ellipticities of the incident RCP and LCP beams are 97.5 % and 97.3 %. The polarization camera was set in the direction normal to the object plane. The distance between the CMOS camera and the object plane was set at 300 mm. To eliminate any reflected light from the sample surfaces, we projected polarized light from a direction of 15 deg from the normal of the object plane. Black paper (T743-1.0; Thorlabs Inc.) was placed behind the samples to ensure that the backscattered light from the black paper was imaged on the CMOS camera. To compare the proposed PPPI technique with conventional PI under unpolarized light (UPL) illumination, we also measured the polarization image after replacing the set composed of the LD, the MEMS mirror, the P, and the QWP with an LED (M970L4; Thorlabs Inc.) with a central wavelength of 980 nm, a bandpass filter (FB980-10; Thorlabs Inc.), a collimating lens, and a rectangular aperture. The rectangular aperture was used to illuminate a rectangular area using unpolarized light. The illumination angle for this unpolarized light was also set at 15 deg. We also measure the accuracy of our system to reconstruct polarization information. Results are shown in Supplementary information.

## Supplementary Information

Supplementary Information.

## Data Availability

The datasets generated during and/or analyzed during the current study are available from the corresponding author on reasonable request.
