# Supplementary material for: Polarization-probe polarization-imaging system in near-infrared regime using a polarization grating
Source: Sci Rep. 2022 Sep 10;12:15268. doi: 10.1038/s41598-022-19536-3 (PMC9464212; doi:10.1038/s41598-022-19536-3)
Supplement: Supplementary file 1 — Supplementary Information. [file 41598_2022_19536_MOESM1_ESM.docx]

**Supplementary information**

**Polarization-probe polarization-imaging system in near-infrared regime using a polarization grating**

Moritsugu Sakamoto^1,4,*^, Huynh Thanh Nhan^1^, Kohei Noda^1,4^, Tomoyuki Sasaki^1,4^, Masayuki Tanaka^2,4^,

Nobuhiro Kawatsuki^3,4^, and Hiroshi Ono^1,4^

^1^Department of Electrical, Electronics, and Information Engineering, Nagaoka University of Technology,

1603-1 Kamitomioka, Nagaoka, Niigata 940-2188, Japan

^2^OPT Gate Co., Ltd., 1-16-12, Shibamata, Katsushika-ku, Tokyo, 125-0052, Japan

^3^Department of Applied Chemistry, University of Hyogo, 2167 Shosha,

Himeji, Hyogo 671-2280, Japan

^4^CREST, Japan Science and Technology Agency, Chiyoda-ku, Tokyo 102-0076, Japan

To show the accuracy of our system, we measured 0deg linearly polarized image by passing LED light scattered from the white sheet through the linear polarizer whose transmission axis is aligned at 0 deg. Since linear polarizer has extinction ratio up to 10^4^, the generating polarization signal should purely 0 deg linearly polarized light. Schematic experimental setup and captured images are shown in Fig. S1 (a) and (b). Reconstructed polarization images ($P$, $\varepsilon$, and ) are shown in Fig. S1. From these results, our system has measurement error of ±5deg for $\varepsilon$ and . We consider that these errors are mainly caused by the miss-alignment and retardation accuracy of LCRs. Also, the uniformity of PG may affect the accuracy of reconstruction. In the future work, we will try to improve the accuracy of our system.


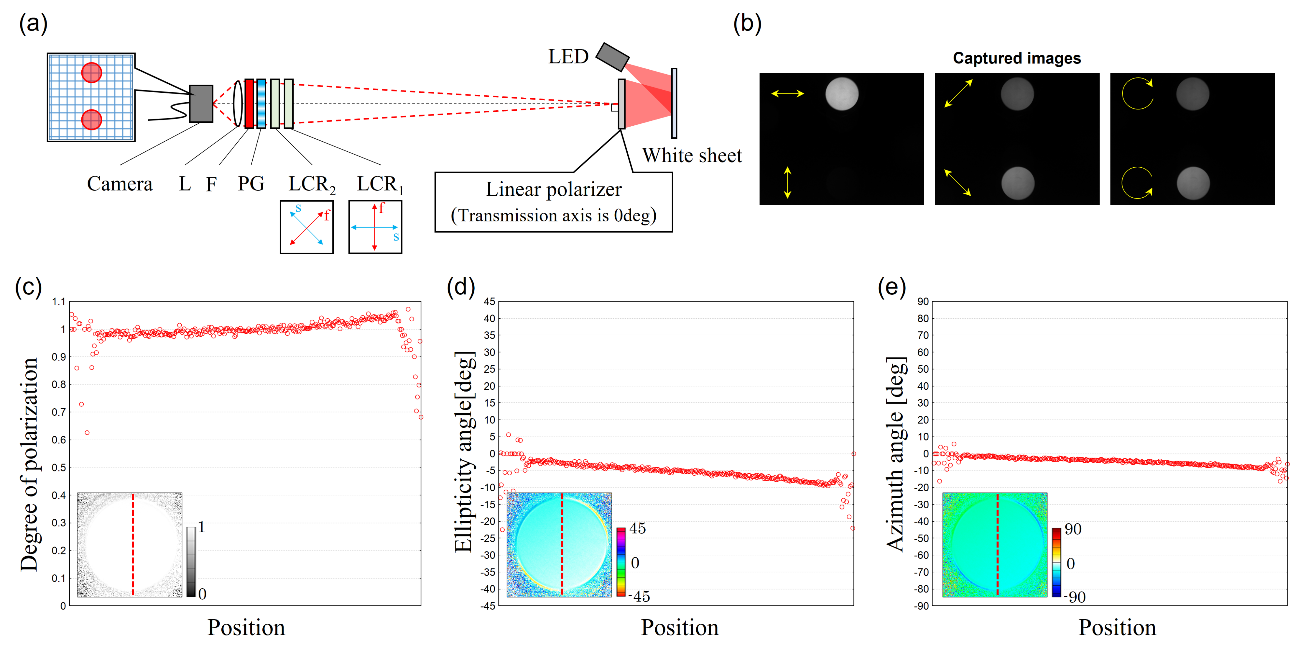


FIG. S1. (a) Schematic of experimental setup for measuring the accuracy of polarization reconstruction. (b) Captured images of the linearly polarized light output from the linear polarizer whose transmission axis is aligned at 0 deg. Reconstructed images of (c) DoP, (d) ellipticity angle, and (e) azimuth angle. Graph shows two-dimensional data along the red dashed line of each reconstructed image.
